# Continuous glucose monitoring in patients with type 2 diabetes on hemodialysis

**DOI:** 10.1007/s00592-021-01699-6

**Published:** 2021-03-20

**Authors:** Maurizio Gallieni, Cristina De Salvo, Maria Elena Lunati, Antonio Rossi, Francesca D’Addio, Ida Pastore, Gianmarco Sabiu, Roberta Miglio, Gian Vincenzo Zuccotti, Paolo Fiorina

**Affiliations:** 1 Department of Biomedical and Clinical Sciences “Luigi Sacco”, Università Di Milano, Milano, Italy; 2 Nephrology and Dialysis Unit, ASST Fatebenefratelli Sacco, Milano, Italy; 3 Division of Endocrinology, ASST Fatebenefratelli-Sacco, Milan, Italy; 4 International Center for T1D, Pediatric Clinical Research Center Romeo Ed Enrica Invernizzi, Department of Biomedical and Clinical Sciences “L. Sacco”, Università Di Milano, Milan, Italy; 5 Pediatric Clinical Research Center Romeo Ed Enrica Invernizzi, Department of Biomedical and Clinical Sciences “L. Sacco”, Università Di Milano and Pediatric Department, Buzzi Children’s Hospital, Milan, Italy; 6 Nephrology Division, Boston Children’s Hospital, Harvard Medical School, Boston, MA USA

**Keywords:** Diabetes mellitus, Continuous glucose monitoring, Hemodialysis, Chronic kidney disease

## Abstract

Diabetic kidney disease is the leading cause of end-stage kidney disease in high-income countries. The strict control of glycemic oscillations is the principal therapeutic target, but this could be hard to achieve in uremic patients due to their unpredictable insulin sensitivity. Currently, the evaluation of the glycemic profile relies on serum markers (glycated hemoglobin HbA1c, glycated albumin, and fructosamine), capillary glucose blood control (self-monitoring of blood glucose), and interstitial glucose control (continue glucose monitoring). We conducted a systematic review of published articles on continue glucose monitoring in hemodialysis patients with type 2 diabetes, which included 12 major articles. Four studies found significant fluctuations in glucose levels during hemodialysis sessions. All studies reported a higher mean amplitude of glucose variations on the hemodialysis day. Three studies agreed that continue glucose monitoring is better than glycated hemoglobin in detecting these abnormalities. Moreover, continue glucose monitoring was more accurate and perceived as easier to use by patients and their caregivers. In patients with type 2 diabetes on hemodialysis, glucose levels show different variation patterns than the patients on hemodialysis without diabetes. Considering manageability, accuracy, and cost-effectiveness, continue glucose monitoring could be the ideal diagnostic tool for the patient with diabetes on hemodialysis.

## Introduction

The long-term metabolic complications of type 2 diabetes (T2D), a rising epidemic [1], include macrovascular and microvascular disorders [2–7], which subsequently induce damage to multiple systems and organs, such as cardiovascular dysfunction and renal impairment [8]. Macrovascular complications include stroke, cardiovascular, and peripheral artery disease. Microvascular diseases comprehend neuropathy, retinopathy, and diabetic kidney disease (DKD) [9]. DKD affects approximately 25% of patients with T2D and is considered the principal cause of end-stage kidney disease in high-income countries [10]. Genetic variability, lifestyle, and diet impact the occurrence of DKD. Notably, the health system organization in the individual country is also a relevant factor [11]. Achievement and maintenance of optimal glycemic control is the principal therapeutic strategy to delay DKD progression as both hypo- and hyperglycemia may exert a negative effect [12]. Intensive treatment regimens can bring to hypoglycemic episodes, which can be hazardous for specific groups of patients, such as those affected by DKD [13]. Patients with diabetes and end-stage kidney disease are at high risk of developing coronary, cerebrovascular, and peripheral vascular disease. These complications are the leading causes of death among end-stage kidney disease patients [5, 14]. Also, numerous abnormalities in the hemostatic system as well as in insulin sensitivity have been described in diabetic patients with end-stage renal disease that may play a critical role in increasing the rate of death in this patient’s population [15–19]. Glycemic control has emerged to be crucial in this population to improve clinical outcomes and significantly reduce cardiovascular risk and mortality. However, proper blood glucose control is challenging in end-stage kidney disease because of the abnormal tissue sensitivity to insulin [20, 21]. Furthermore, there is a lack of precision by classical markers of glycemic control (i.e., HbA1c and fructosamine) due to analytical interferences, to shortened half-life of red blood cells and to abnormal albumin level [22]. Several techniques are used in patients on hemodialysis for glucose measurement at home and during dialysis; indeed, self-monitoring of blood glucose is still the most used 1 and the traditional method to routinely monitor blood glucose [23]. Unfortunately, self-monitoring of blood glucose does not provide continuous measurements, thus making the evaluation of the patient's glycemic profile incomplete, resulting in glycemic fluctuations. Since 1999, continuous glucose monitoring (CGM) is increasingly being used because it allows real-time blood glucose evaluation. The device records up to 280 measurements per day (generally every 1–5 min) and has a transmitter that stores or sends the values (generally every 5–15 min) to a receiver. The glucose sensor reading calibration requires capillary blood sugar measurement with a traditional glucose meter (two/four tests per day) [24]. The glucose concentration in the interstitial fluid generally closely approximates that of blood glucose, particularly when glucose concentrations are stable [25], while during periods of rapid glucose change, the lag can be greater. The mean absolute relative difference (MARD) has become popular for evaluating the overall accuracy of the CGM [26]. There are two types of CGM: (i) RT-CGM allows real-time measurement of blood glucose, and (ii) r-CGM (retrospective CGM) allows to record a series of measures but real-time data results are not available directly to patients, and the physician will have access to them at the end of the monitoring period. In 2014, a new category of device was introduced, the flash glucose monitoring system (FGM) that allows the obtainment of glucose values instantly by scanning the glucose sensor with the reader, producing real-time on-demand glucose data. The aim of this review was to analyze the available literature on the use of continuous glucose monitoring in patients with type 2 diabetes on hemodialysis and demonstrate whether CGM may represent a reliable tool to reduce glucose variability and improve diabetes management in that fragile patient’s population.

## Methods

### Sources

We used MEDLINE (1976–present) and Cochrane Library as the primary sources of literature search. We considered only human subjects and the English language. We undertook a literature review searching for multiple pairs of keywords, including “continuous glucose monitoring and hemodialysis,” “continuous glucose monitoring and end-stage renal disease,” and “continuous glucose monitoring and dialysis.” We considered any study, including case reports, observational studies, and RCT. Our research with key relevant search words produced 552 results. We excluded duplicates. We excluded studies on pediatric patients. The majority of the studies reviewed included patients with type 2 diabetes. There were only a few articles which had a population of patient with type 1 diabetes and secondary diabetes. With regard to kidney replacement therapy, hemodialysis, and peritoneal dialysis, only three studies analyzed a population of peritoneal dialysis patients, and one of them was a case report [27]; therefore and because of different daily glucose profile, peritoneal dialysis studies were not included in the analysis. Moreover, one article utilized CGM to compare the efficacy of two oral glucose-lowering drugs [28]. After reviewing all studies’ titles and abstracts, we selected 12 of them as appropriate for full-text reading and further analysis [24, 29–39]. Table 1 shows baseline patient’s characteristics, while studies design, the outcome of interest, and findings are reported in Table 2. The main outcome in 10 out of the 12 studies analyzed was to monitor the glycemic profile and assess blood glucose variability in hemodialysis patients. In one study the main endpoint was to evaluate the effectiveness of glycated albumin in monitoring the glycometabolic control as compared to glycated hemoglobin in hemodialysis patients [30]. In one study the main endpoint was to assess whether glycemic monitoring may guide therapeutic decision on insulin treatment in hemodialysis patients [36].

**Table 1:** Baseline patients' characteristics of the selected studies (*n* = 12)

References		Patient’s characteristics						
		No. of patients	Age (years)	Sex		Types of diabetes	Diabetes duration (months)	Dialysis duration (months)
				Male	Female			
Gai et al. [24]		12	62 ± 14	9 (75%)	3 (25%)	II	39.6 (1.9−125.4)	21.2 (2.2−41.7)
Jin et al. [31]	ESDKD	36	62 ± 13	29 (79%)	7 (21%)	II	156 ± 84	/
	ESKD	10	65 ± 13	8 (80%)	2 (20%)	/	0 ± 0	/
Jung et al. [33]		9	67 ± 9	/	/	II	288 ± 108	/
Képénékian et al. [29]		27	66 ± 9	9 (32%)	19 (68%)	II	273 ± 117	43 ± 30
Chantrel et al. [29]		33	66 ± 8	19 (58%)	14 (42%)	II	276 ± 132	46 ± 31
Mirani et al. [36]		12	62 ± 10	7 (58%)	5 (42%)	II	180 ± 96	27.6 ± 15.6
Riveline et al. [38]	ESDKD	19	64 ± 10	8 (42%)	11 (58%)	II	252 ± 132	24 (13−35)
	Non-HD	39	65 ± 6	25 (64%)	14 (36%)	/	204 ± 84	/
Kazempour-Ardebili et al. [34]		17	61 ± 9	13 (76%)	4 (23%)	II	225 ± 91	48 ± 31.2
Divani et al. [30]		37	62 ± 17	20 (54%)	17 (46%)	/	/	37.0 ± 16.9
Mori et al. [37]		1	68	1 (100%)	0 (0%)	/	492	216
Joubert et al. [32]		15	61 ± 15	8 (53%)	7 (47%)	I and II	230 ± 91.2	78 ± 83
Yajima T et al. [39]		13	63.5 ± 11.3	11 (85%)	2 (15%)	II	/	7.3 (4.3−28.4)

*ESDKD* end-stage diabetic kidney disease, *ESKD* end-stage kidney disease

**Table 2:** Outcomes of interests and findings of the selected studies (*n* = 12)

References	No. of pts	Design	Arms	Type of CGM	Follow-up (Days)	Outcome of interest	Findings
Gai et al. [24]	36	Perspective observational	1	r-CGM	6	Glycemic monitoring in HD	Glycemic fluctuations and hypoglycemia during HD
Jin et al. [31]	46	Perspective observational	2	r-CGM	3	Glycemic monitoring in HD	Glycemic fluctuations during HD
Jung et al. [33]	9	Perspective observational	1	r-CGM	6	Glycemic monitoring in HD	No glycemic fluctuations but increased risk of hypoglycemia during HD
Chantrel et al. [29]	33	Perspective observational	1	rt-CGM	90	Glycemic monitoring in HD	Glycemic fluctuations and hypoglycemia during HD
Mirani et al. [36]	12	Perspective observational	1	r-CGM	2	Glycemic monitoring in HD	Large glycemic fluctuations during HD
Riveline et al. [38]	58	Perspective observational	2	r-CGM	4	Glycemic monitoring in HD	No glycemic fluctuations during HD
Kazempour-Ardebili et al. [34]	17	Perspective observational	1	r-CGM	2	Glycemic monitoring in HD	Glycemic fluctuations and hypoglycemia during HD
Divani et al. [30]	37	Perspective observational	1	r-CGM	7	Validity/accuracy of CGM in HD	CGM is accurate in glycemic monitoring
Képénékian et al. [29]	27	Perspective observational	1	rt-CGM	90	Improving DM therapy in HD	Improved glycemic control with CGM
Joubert et al. [32]	15	Perspective observational	1	r-CGM	90	Improving DM therapy in HD	Large glycemic fluctuations during HD and improved glycemic control with CGM
Mori et al. [37]	1	Case report	/	rt-CGM	2	Glycemic monitoring in HD	Glycemic fluctuations and hypoglycemia during HD
Yajima T et al. [39]	13	Perspective observational	1	r-CGM/ FGM	2	Glycemic monitoring in HD	Accuracy of FGM in HD

*PTS* patients, *HD* hemodialysis, *CGM* continuous glucose monitoring, *ESDKD* end-stage diabetic kidney disease, *ESKD* end-stage kidney disease

## Results

### CGM and hypoglycemic risk in hemodialysis

The augmented risk of TBR (time below range) during dialysis days and a greater reliability of CGM, compared to glycated hemoglobin, has been hypothesized by Kazempour-Ardebili et al. [34]. The study showed average 24-h glucose values significantly higher during non-dialysis days than dialysis days independent of energy intake, with a higher risk of TBR within 24 h of dialysis. The difference between average 24-h glucose levels for the dialysis-free day to the dialysis day ranged from −2.1 to 10.4 mmol/l (−38 to 187 mg/dl). Similar conclusions were obtained by Gai et al. [24]. They analyzed the utility of CGM as an extended glycemic control method in 12 patients with T2D on hemodialysis. During dialysis, serum glucose diminished, while TAR (time above range) episodes were more frequent in the post-dialysis period. CGM was an excellent method to detect these fluctuations. The association of hemodialysis treatment and TBR risk was evaluated also by Jung et al. [33]. In 9 patients with T2D on hemodialysis using CGM, daily glucose fluctuations were not associated, while hypoglycemia was, with hemodialysis. Also, Riveline et al. [38] evaluated the clinical performance of CGM in patients with T2D on hemodialysis. The study compared 19 patients with T2D on hemodialysis with 39 patients with T2D not on hemodialysis. The comparison between hemodialysis day and non-hemodialysis days differed remarkably in the first 3 h of dialysis. During this time, the mean glucose concentration was significantly lower than in non-hemodialysis days, although only two patients had intra-dialytic hypoglycemia (< 70 mg/dl). Recently, a case report by Mori et al. [37] described a patient with T2D on hemodialysis monitored with CGM, in which several TBR episodes were recorded during hemodialysis session. To summarize, there is a general agreement that CGM reduces TBR episodes in patients with T2D on hemodialysis.

## CGM and glucose variability in hemodialysis

Blood glucose variability in patients with T2D on hemodialysis was quite extensively investigated. Mirani et al. [36] studied 12 patients with T2D on hemodialysis for two days, including one hemodialysis day and the following non-hemodialysis day. The mean 24-h glycemic value and the mean amplitude of glycemic excursions (MAGE) were significantly higher in the hemodialysis day than the non-hemodialysis day. The study also showed a direct correlation between the mean glucose concentration and glycated hemoglobin, whereas no association existed between the glucose profile variability and glycated hemoglobin. Jin et al. [31] aimed to characterize the blood glucose fluctuations during hemodialysis with CGM. Glycemic variability was assessed by measuring the MAGE. Forty-six patients were divided into 2 groups: 36 patients with T2D on hemodialysis and 10 patients without T2D on hemodialysis. They found out that the first group had larger and more significant glycemic fluctuations. Moreover, glycated hemoglobin was inaccurate since it did not reflect the correct blood glucose variability during an extended period. Similar conclusions have been reported by Chantrel et al. [29]. They analyzed 33 patients with T2D on hemodialysis in insulin treatment with 3 CGM sessions of 48 h each, including a dialysis session, over 3 months. CGM results were analyzed during and after hemodialysis and in other different day periods according to meals. Mean glucose values, MAGE, and coefficient of variation (%) improved, whereas the frequency of TBR was higher during hemodialysis sessions. Moreover, significant differences were observed in glucose levels before and 2 h after breakfast. Patients with T2D on hemodialysis are subject to the high variability of glucose profiles, and standard laboratory assays would miss such variations. Also, the use of hypoglycemic drug may enhance glucose variability and increase the risk for hospitalization in these patients [40]. In conclusion, CGM would be a handy tool to detect such variations and manage these complex patients properly.

## CGM and improvement of diabetes management in hemodialysis

The role of CGM as a tool to manage the insulin regimen in patients with T2D on hemodialysis was tested by Képénékian et al. [35]. CGM was applied for 54 h at baseline and a 3-month follow-up in 28 patients to adapt insulin therapy to the CGM values. After 3 months, patients demonstrated a significant reduction of glycated hemoglobin and CGM glucose values, with no episodes of severe hypoglycemia. Furthermore, patients experienced less episodes of TAR with reduction in insulin requirements. The study did not focus on analyzing glycemic profiles on hemodialysis days compared with non-hemodialysis days, even though it underscored the absence of intra-dialytic TBR episodes. Képénékian et al. concluded that the CGM-adapted insulin regimen could be a useful tool for managing diabetes in patients on hemodialysis. Also, the *DIALYDIAB pilot study* [32] aimed to analyze the use of CGM to detect glucose fluctuations in patients with diabetes on hemodialysis. Fifteen patients were enrolled and were studied for the first period by self-monitoring blood glucose 3 times a day and then for the second period by CGM. They observed that during CGM monitoring, treatment changes took place more frequently, resulting in better blood glucose control and fewer TBR episodes. Finally*,* Divani et al. [30] compared various monitoring blood glucose methods over 7 days in hemodialysis patients using CGM. The study concluded that 7-day-long CGM is better to assess poor glycometabolic control as compared to glycated hemoglobin. This study also underscored the accuracy of CGM. Regarding the accuracy of FGM in hemodialysis patients, Yajima et al. [39] studied 13 uremic patients undergoing simultaneously FGM, CGM and self-monitoring blood glucose during hemodialysis and non-hemodialysis days. Their conclusions showed that the use of FGM may be clinically relevant in this population, but MARDs for TBR and TIR were significantly higher than MARD for TAR. Moreover, MARD for FGM was significantly higher than for CGM, in both hemodialysis and non-hemodialysis day, while MARD for CGM on hemodialysis day was significantly higher than that on non-hemodialysis day, due to the higher glycemic excursion as reported by Jin et al. [31]. More detailed studies are needed to evaluate comparison between FGM or CGM during and after hemodialysis.

## Conclusions

The review of the literature surrounding the use of CGM in hemodialysis revealed few important messages. First and foremost, the mean amplitude of glucose variations was higher in the hemodialysis days than in those without hemodialysis. The glucose concentration in the dialysis fluid, hemodialysis sessions duration, hemofiltration of drugs, and the time frame between meals and hemodialysis sessions are probably the factors responsible for the observed differences. Second, the use of CGM may reduce hypoglycemic episodes which appeared increased during hemodialysis. The conclusion is that CGM would be a useful tool in detecting these abnormalities and in improving the management of diabetes [32]. All the studies identified CGM as an appropriate and reliable tool to detect glycemic variations and hypoglycemic episodes in this population, particularly on the hemodialysis day. Furthermore, several studies have shown that CGM is much better perceived by patients and their caregivers, who appreciated the possibility of easily accessing blood glucose data [41]. The device provides trend arrows that add context to each glucose reading; this has a critical impact on insulin dosing decision and hypoglycemia prevention. Only 1 study assessed CGM's cost-effectiveness, but it considered only patients with type 1 diabetes and anyway CGM was cost-effective [42]. In patients without chronic kidney disease (CKD), the accuracy of the CGM may vary depending on the blood glucose concentration and the rate of blood glucose change [43, 44]. A comparison of the accuracy of FGM and CGM in non-CKD patients recently demonstrated that both systems perform safely and efficiently but accuracy of the CGM sensor appear higher across all glucose values except in hypoglycemia [45]. This review has several limitations, including the limited number of patients and the short-term follow-up. Despite the prospective nature, most of the studies remained observational studies and almost all of them without a comparison arm. Besides, not all studies focused on the same parameters, some observed glycemic variations, while others focused on the modulation of diabetes treatment based on the CGM device results. In conclusion, the use of CGM in patients with diabetes on hemodialysis ensures the improvement in glucose control and reduces the risk of hypoglycemia, especially in adults with type 1 diabetes, still experiencing suboptimal glycemic control. CGM could improve patients' management and quality of life and it is cost-effective.

## Data Availability

Not applicable.
